# Multisite Venovenous Extracorporeal Membrane Oxygenation in Pediatric Patients Under 15 kg: Three-Center, Analysis of Surgical Versus Percutaneous Approach and Thrombosis Risk, 2017–2024

**DOI:** 10.1097/PCC.0000000000003858

**Published:** 2025-11-07

**Authors:** Cecilia Korb, Polyxeni Mantziari, Janos Schnur, Veronika Maraczi, Barbara Szasz, Jon Lillie

**Affiliations:** 1 Paediatric Intensive Care, Evelina London Children’s Hospital, Guy’s and St Thomas’ NHS Foundation Trust, London, United Kingdom.; 2 Paediatric Intensive Care, Heim Pal National Paediatric Institute, Budapest, Hungary.; 3 Division of Neonatology, Pediatric Center, Semmelweis University, Budapest, Hungary.; 4 Centre for Critical Illness Research, King's College London, London, United Kingdom.

**Keywords:** adverse effects, cannula, extracorporeal membrane oxygenation, neonate, respiratory failure

## Abstract

**Objectives:**

An increasing number of pediatric centers use the femoral vein in neonates and smaller children to provide venovenous multisite (VVMS) extracorporeal membrane oxygenation (ECMO), but there are no studies comparing surgical vs. percutaneous approaches. We investigated the thrombosis risk associated with VVMS, comparing outcomes between the surgical and percutaneous approach.

**Design:**

Retrospective data analysis.

**SETTING:**

Evelina London Children’s Hospital, Heim Pal Hospital and Semmelweis University, 2017–2024.

**Patients:**

We included 58 neonatal and pediatric patients weighing less than 15 kg, supported with VVMS ECMO for respiratory disease.

**Interventions:**

None.

**Measurements and Main Results:**

We collected patient and venovenous ECMO details. Thrombosis of cannulated vessels, as determined by a pediatric radiologist via follow-up vascular ultrasound, was the primary outcome. In total, 58 patients received VVMS, survived and were assessed for thrombosis. There were 34 surgically cannulated patients with median (interquartile range [IQR]) weight 3.6 kg (IQR, 2.8–7 kg) vs. 24 percutaneously cannulated patients (median weight, 8 kg [IQR, 3.7–12.3 kg]) who were cannulated by pediatric anesthetists or intensive care physicians. Surgical placement of cannulas, in comparison with percutaneous placement, was associated with greater odds of thrombosis of internal jugular and femoral veins (odds ratio, 37; 95% CI, 7–266), although the surgical group were younger and of lower weight (*p* < 0.05). For any given weight, percutaneously placed cannulas were smaller than those placed surgically, yet they still provided adequate ECMO flow.

**Conclusions:**

This retrospective case series of VVMS in neonates and small children (< 15 kg) shows that surgical cannulation was associated with greater odds of vascular thrombosis at vessel sites, but a potential confounder may be that the surgical group were younger and of smaller weight. Overall, for any given weight, percutaneous cannulas were smaller than those placed surgically.

**RESEARCH IN CONTEXT**

- Venovenous multisite (VVMS) extracorporeal membrane oxygenation (ECMO) is increasingly used for neonates and small children to avoid the neurologic risk of venoarterial ECMO and the technical difficulties of dual lumen cannulas.
- Surgical cannulation technique is most used but is associated with common femoral vein thrombosis, albeit with no associated clinical significance at 1-year follow-up.
- In this three-center, retrospective report, we have compared our experience of percutaneous vs. surgical technique for VVMS ECMO to examine thrombosis risk.

**AT THE BEDSIDE**

- In our three-center, retrospective cohort (2017–2024) of neonates and small children, we have found that surgical cannulation for VVMS ECMO is associated with greater odds of venous thrombosis compared with the percutaneous technique.
- For a given weight, VVMS cannulas placed percutaneously were smaller than those inserted surgically.
- Adequate VVMS ECMO flows were delivered with 10-Fr access/8-Fr return for neonates and 12-Fr access/10-Fr return for older infants and children (< 15 kg), which are smaller than the 2022 Extracorporeal Life Support Organization Handbook recommendations.

Neonatal and pediatric patients with respiratory failure requiring extracorporeal membrane oxygenation (ECMO) have been supported with both venoarterial and venovenous ECMO. Although venoarterial ECMO is associated with a higher risk of mortality and neurologic complications (1–3), venovenous ECMO rates in younger patients have declined due to concerns regarding suitability of dual lumen cannulas for neonates (4). In more recent years, venovenous multisite (VVMS) ECMO, accessing the right internal jugular (RIJ) vein and returning via the common femoral vein (CFV), has been increasingly reported in case series as well as the Extracorporeal Life Support Organization (ELSO) registry (2000–2019), with successful cannulation of patients down to less than 2 kg (5–7). Also, this technique avoids difficulties associated with dual lumen cannulas and the neurologic risks associated with venoarterial ECMO.

Despite the success of VVMS, a concern in neonates and small infants is complete occlusion of the CFV on follow-up (5). Furthermore, although surgical cannulation in neonatal and pediatric venovenous ECMO is the usual practice, percutaneous ultrasound-guided cannulation in neonates and smaller pediatric patients is considered an option in the 2020 ELSO neonatal respiratory failure guidelines (8). Since there are no studies comparing surgical and percutaneous cannulation techniques regarding thrombosis rates, we aimed to review our VVMS experience across our three ECMO centers. We have also explored factors associated with any differences observed.

## METHODS

The protocol for this retrospective cohort study titled “Thrombosis Risk in Multi-Site Venovenous ECMO Cannulation” was first approved with a waiver of consent by the Evelina London service evaluation and audit team (our local equivalent of an institutional review board [IRB]) on November 14, 2024, project 16798. Besides the Evelina London Children’s Hospital (United Kingdom), two other hospital centers collaborated in this project: the Heim Pal Children’s Hospital and the Semmelweis University Pediatric Center in Budapest, Hungary. Both hospitals in Hungary gained local IRB approval for collecting and sharing retrospective data covering the period from January 2017 to March 2024. All three centers have collaborated in the past with shared teaching and similar anticoagulation protocols (**Supplementary Digital Content**, https://links.lww.com/PCC/C669). All research procedures were carried out in accordance with local regulations, and followed the ethical standards of the Helsinki Declaration of 1975, amended 2024.

### Cannulation Procedures and Patient Collection

Surgical cannulation was undertaken by Evelina London Children's Hospital and performed by the cardiothoracic surgical team at the bedside with cutdown vessel exposure and cannulation under direct vision as described in a previous case series (5). Cannulas were preferentially tunneled under the skin so that they entered the skin distally to the surgical incision site. Cannulas were secured to the vessel using purse string sutures and additionally sutured to the skin. Percutaneous cannulation was favored by Budapest centers and performed by physicians who were pediatric intensivists or anesthetists. There were always at least two clinicians undertaking the procedure who were both proficient in central venous cannulation using ultrasound. At least one of the operators was a consultant. Cannulas were placed using the Seldinger technique under ultrasound guidance. All operators undertook bespoke training before cannulation, which included familiarization with ECMO cannulas, introducers, wires, and attaching cannulas to the ECMO circuit using a wet join.

The inclusion criteria for study were neonatal and pediatric patients with a weight of less than 15 kg who required VVMS ECMO for respiratory support. We excluded patients who did not undergo follow-up ultrasound scan (USS) to assess for thrombosis. The variables included in our analyses were: weight, age, indication for ECMO, size of cannula in French gauge (Fr), location (vessel) of cannulas, complications at cannulation, duration of ECMO run, ECMO flows (mL/min and mL/min/kg) and oxygen saturations after 24 hours of ECMO, and USS evidence of thrombosis at follow-up.

### Outcomes and Statistics

The primary outcome was thrombosis of vessel after decannulation as diagnosed by vascular USS performed by a pediatric radiologist or angiologist while an inpatient at the ECMO center. Equipment used at each center was comparable, including: Philips (Eindhoven, The Netherlands) Affiniti in Evelina London Children’s Hospital; Philips Epiq in Heim Pal Children’s Hospital; and Samsung (Seoul, South Korea) HS50 in Semmelweis University. The term thrombosis included nonocclusive thrombosis, occlusive thrombosis, and thrombosis with extension to neighboring vessels.

Descriptive statistics were used to summarize data and included count, proportions and percentages (%) with 95% CI, mean and sd, and median and interquartile range (IQR), as appropriate. Mann-Whitney *U* equality of populations test was performed to compare characteristics between groups. Results of comparison of proportions are presented as mean difference in percentage with the 95% CI of the percentage difference. Logistic regression with odds ratio (OR) and 95% CI was used for presenting the primary outcome results. Post hoc analysis was performed to illustrate the distribution of cannula sizes against weight for both cannulation techniques using a linear model, and to assess the interaction between cannulation technique and weight and cannula size using the Wald interaction test. Statistical analysis was performed using Stata 17 (StataCorp LLC, College Station, TX, 2021).

## RESULTS

In the 2017–2024 cohort, across our three centers, there were 64 neonatal and pediatric patients who underwent VVMS. Six of these patients were excluded because they had not had USS assessment for thrombosis: four patients died before follow-up vascular USS, one patient was transferred to another ECMO center before decannulation, and one patient did not undergo follow-up vascular USS. Therefore, 58 patients were included in the final analysis with 34 of 58 (59%) cannulated surgically and 24 of 58 (41%) cannulated percutaneously (**Table 1**). On comparing the surgical vs. percutaneous groups, there was an association between technique and patient age/weight (median age of 1 mo [IQR, 0–9 mo] vs. 10 mo [IQR, 0–30 mo]; *p* < 0.05 and median weight of 3.6 kg [IQR, 2.8–7.0 kg] vs. 8 kg [IQR, 3.7–12.3 kg]; *p* < 0.05).

**TABLE 1.** Population Demographics and Extracorporeal Membrane Oxygenation Characteristics

Variable	All Patients (*n* = 58)	Percutaneous (*n* = 24)	Surgical (*n* = 34)
Age (mo)	4 (0–15)	10 (0–30)	1 (0–9)
Weight (kg)	4.5 (3.0–8.9)	8 (3.7–12.3)	3.6 (2.8–7.0)
Indication for extracorporeal life support			
Acute respiratory distress syndrome	3/58 (5%)	3/24	0/34
Air leak	5/58 (9%)	4/24	1/34
Alveolar disease	3/58 (5%)	1/24	2/34
Congenital diaphragmatic hernia	1/58 (2%)	0/24	1/34
Interstitial lung disease	3/58 (5%)	3/24	0/34
Lower respiratory tract infection	26/58 (45%)	8/24	18/34
Meconium aspiration syndrome	11/58 (19%)	3/24	8/34
Sepsis	6/58 (10%)	2/24	4/34
Flow at 24 hr (mL/min)	505 (383–1006)	835 (425–1338)	435 (360–850)
Flow at 24 hr (mL/min/kg)	122 (106–136)	119 (99–129)	126 (115–139)
Oxygen saturation at 24 hr^a^ (%)	94 (90–98)	92 (86–95)	96 (93–99)
Duration of extracorporeal membrane oxygenation run (d)	7 (5–13)	7 (5–17)	7 (4–12)

a Oxygen saturation defined as pre-ductal in neonatal patients.

Median and interquartile ranges for continuous data and percentages shown for categorical data.

In the surgical group, one of 34 patients (95% CI, 0.5–14.9%) had a complication at cannulation with a perforation of the right atrium from the RIJ access cannula, causing a pericardial effusion, which was surgically evacuated without compromise to the patient. In the percutaneous group, there was one complication (1/24 patients [95% CI, 0.7–20.2%]) with kinking of the RIJ wire after vessel dilatation, which was removed with surgical assistance. The RIJ was then successfully cannulated percutaneously. Regarding oxygen saturation at 24 hours, there was an association with cannulation technique, with higher median saturation in the surgical vs. percutaneous groups (96% [IQR, 93–99%] vs. 92% [IQR, 86–94%]; *p* < 0.05; Table 1).

**Table 2** summarizes the thrombosis data. The commonest vessels used for access and return cannulation were the RIJ and right CFV respectively in both groups. On comparing thrombosis frequency of cannulated vessels in the surgical vs. percutaneous groups, we have 29/34 vs. 4/24 (mean difference, 68.3%; 95% CI, 43.6–81.3; *p* < 0.0001). After logistical regression, surgical cannulation in comparison with the percutaneous technique was associated with greater odds of thrombosis (OR, 37 [95% CI, 7–266]).

**TABLE 2.** Access and Return Cannula Location and Associated Thrombosis According to Cannulation Technique

Site of Cannula	Percutaneous (*n* = 24)		Surgical (*n* = 34)		
	Vessel	Thrombosis	Vessel	Thrombosis	
Access					
Left brachiocephalic	1	1/1	0	Not applicable	
Left internal jugular	2	0/2	0	Not applicable	
Right internal jugular	21	1/21	34	20/34	
Return					
Left femoral vein	4	1/4	4	2/4	
Right femoral vein	20	1/20	30	25/30	
Total thrombosis	4/48 vessels; 4/24 patients		47/68 vessels; 29/34 patients		Odds ratio, 37 (95% CI, 7–266)

**Figure 1.**
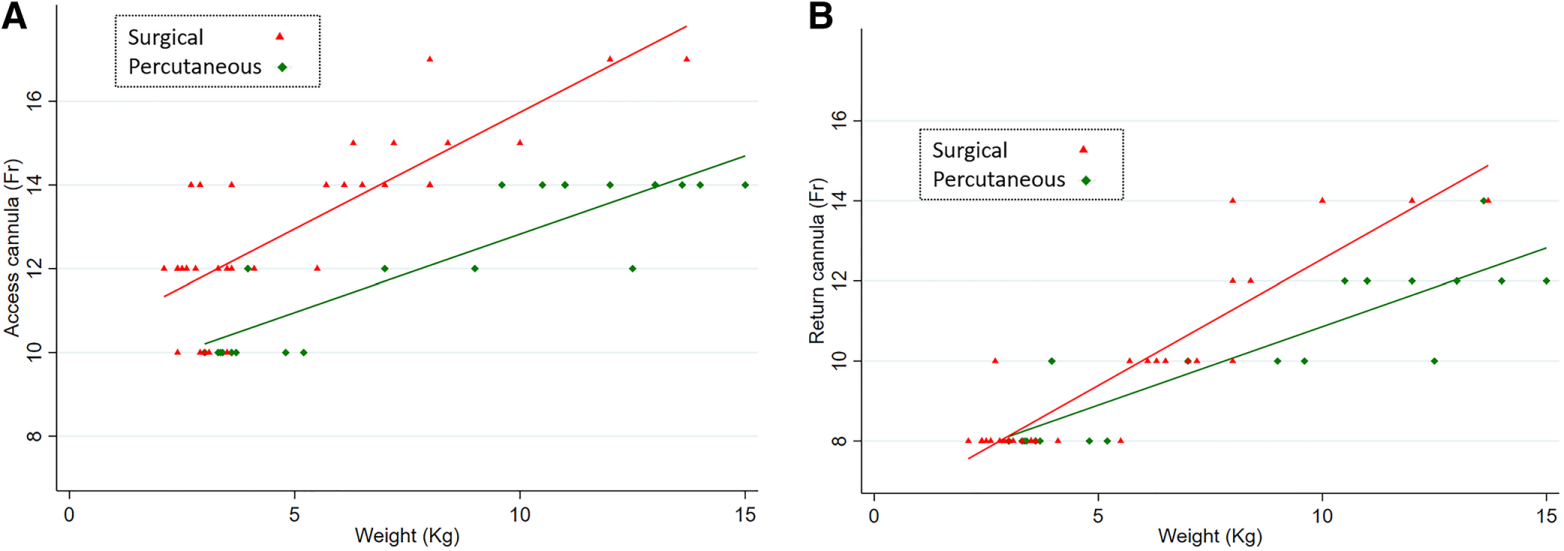
Graphs showing distribution of cannula size against weight. **A**, Access cannula size. **B**, Return cannula size. Comparison of surgical (*red triangles*) and percutaneous (*green diamond*) technique with lines of best fit based on a linear model.

**Figure 1** plots the access and return cannula size by patient weight. The regression lines show that being cannulated percutaneously was associated with use of smaller cannula for weight, in comparison with the surgical group. The associated difference was more pronounced for the access (coefficient, 2.22 [95% CI, 1.58–2.87]; *p* < 0.001) than the return cannula (coefficient, 0.79 [95% CI, 0.23–1.34]; *p* = 0.006). On visual inspection of Figure 1*A*, we see that regarding the access cannulas for a given weight, there is a difference of approximately 2-Fr between 4 and 10 kg body weight. In the return gauge graph (Fig. 1*B*), smaller cannulas are used, and the difference appears apparent when body weight is greater than 9 kg and is of the order of two differences in Fr gauge. Flows delivered at 24 hours with pairs of cannulas used on each patient are summarized in **Table 3**. The most frequently used pair of cannulas were 10-Fr access and 8-Fr return, which delivered a median flow of 398 mL/min.

**TABLE 3.** Access and Return Configuration of Cannulas and Median Flows

Access Cannula	Return Cannula			
	8-Fr	10-Fr	12-Fr	14-Fr
10-Fr	398 (*n* = 15)	—	—	—
12-Fr	392 (*n* = 12)	896 (*n* = 5)	—	—
14-Fr	345 (*n* = 2)	827 (*n* = 7)	848 (*n* = 10)	1590 (*n* = 1)
15-Fr	—	808 (*n* = 2)	990 (*n* = 1)	1200 (*n* = 1)
17-Fr	—	—	—	1498 (*n* = 3)

Flows are described as mL/min.

Median flows delivered at 24 hr for pairs of cannulas (access and return). Number of patients with each pair of cannulas are shown.

## DISCUSSION

In our three-center, retrospective cohort (2017–2024) of patients undergoing VVMS, we have compared associated thrombosis rates by cannulation technique (surgical vs. percutaneous) in neonates and pediatric patients with weight up to 15 kg. First, our data showed that there was an association between cannulation technique and patient size: use of the percutaneous technique was associated with older age and heavier weight. Second, the surgical approach was associated with 37-fold greater odds of vessel thrombosis identified by USS on follow-up. Third, for a given patient weight, practitioners placing percutaneous cannulas used smaller French gauge than those placing cannulas surgically. Last, despite small size of the percutaneous cannulas, adequate ECMO flows were achieved, and comparable with the 2022 ELSO standard (9).

There is paucity of data regarding venous thrombosis and pediatric ECMO cannulation. ELSO database 2000–2019 reports thrombosis in only 23% of venovenous ECMO patients but there is no standard monitoring of vessel thrombosis for centers reporting to this database (7). Thrombosis is likely to be underreported as adult ECMO studies that specifically look for deep vein thrombosis found a prevalence of 46–85% post-decannulation from venovenous ECMO, and a higher frequency is associated with longer ECMO runs and systemic infection (10–12). The two groups in our study had similar ECMO run durations and a small number in each group with documented sepsis, precluding these as confounding factors. Furthermore, other risk factors associated with pediatric deep vein thrombosis, such as malignancy or trauma were not present in either of the studied groups (13, 14).

Our three collaborating centers in the United Kingdom and Hungary have similar anticoagulation policies for patients on ECMO (Supplementary Digital Content, https://links.lww.com/PCC/C669), and we use unfractionated heparin infusion as the anticoagulant of choice. Monitoring is facilitated by anti-factor Xa assay in Evelina Children’s Hospital and Semmelweis University, while activated partial thromboplastin time is used in Heim Pal Children’s Hospital. These practices are considered alike when monitoring unfractionated heparin therapy (15), and they are consistent with the 2024 Pediatric ECMO Anticoagulation Collaborative consensus (16–18). Also, across our ECMO centers, assessment of thrombosis is similar and consists of vascular USS performed and interpreted by a pediatric radiologist or angiologist, using equipment of comparable technology and accuracy, reducing bias related to different interpretation of the primary outcome. With regards to cannulation, it is important to appreciate that operators were limited to a small number of surgeons or physicians within each center to maximize exposure and gain expertise. For example in one center, one of two pediatric intensive care consultants was present for every cannulation and surgical cannulation was undertaken by a team of four surgical consultants.

In similar weight patients, percutaneously cannulated patients had smaller size ECMO cannulas inserted than those used for the surgical cannulation procedure (Fig. 1). A potential reason for this is that our surgical practitioners have been taught to choose the largest cannula that they think is possible to place in the exposed vessel. The rationale for this is so that the cannula size does not limit ECMO flow but may mean that larger cannulas are placed than are necessary. The lower rate of thrombosis in the percutaneous group may be because the technique allows blood flow around the cannula compared with the surgical technique in which a bigger cannula is secured to the vein with a suture. Using smaller cannulas raises the concern of increased resistance that could limit flow, thereby rendering ECMO inadequate. In our study, median flows at 24 hours were calculated per pair of cannulas, by sizes, and across the whole study population, irrespective of cannulation technique used. Adequate flows for neonatal patients requiring ECMO for respiratory support were delivered with excellent oxygenation via a pair of 10-Fr access and 8-Fr return cannulas. Similarly, a pair of 12-Fr access and 10-Fr return cannulas provided adequate flow for an infant weighing up to 9 kg (Table 3). These are smaller sizes than those advised in the 2022 ELSO handbook but we report real-world VVMS data while their graphs are extrapolated from cannulas used for venoarterial ECMO or older venovenous patients (9). This observation could have important implications for delivery of venovenous ECMO in neonates with congenital diaphragmatic hernia (CDH), which is the commonest indication of neonatal ECMO in the ELSO registry 2000–2019 (19). Neonates with CDH have smaller neck vessels than other neonates (20), so being able to deliver effective flow via a smaller 10-Fr cannula in the RIJ would be beneficial and potentially allow more babies with CDH to receive venovenous ECMO. Percutaneous cannulation using VVMS in a baby with CDH weighing less than 2 kg has been reported in a retrospective case series of 54 neonates, 2017–2021 (6).

There are two main limitations in our retrospective study. First, the numbers were small and we could not control for center effect in the analysis of associations between cannulation technique and thrombosis. That said, our centers have collaborated in developing VVMS and we believe our practices regarding anticoagulation, maintaining good ECMO flow, and identification of thrombosis, are similar. However, there may be some unknown confounder that contributed to risk of thrombosis, for example, we did not compare markers of hemolysis like plasma-free hemoglobin. Second, our cohort was heterogeneous in terms of age and weight, with there being an association between selection for surgical procedure and the patient being of smaller weight and/or younger age. As vessels are smaller in smaller and younger age patients, this issue could contribute to associated greater odds of thrombosis in the surgical group. When evaluating whether smaller patient size could be the “main” factor associated with thrombosis, it is reassuring to note that all the surgically cannulated neonates had occlusion of the femoral vein compared with none of the seven neonates cannulated in the percutaneous group. The sample size is too small for us to be able to control for patient size and weight.

In conclusion, in our three-center, 2017–2024 cohort of neonates and young children (weight < 15 kg) undergoing VVMS for severe respiratory failure, we have compared the association between cannulation technique (surgical vs. percutaneous) and thrombosis. We found that surgical venovenous cannulation was associated with greater odds of vascular thrombosis. However, since the selection for a surgical approach was associated with being small and/or younger age, we cannot exclude these as potential confounding factors. Last, we also showed that the cannulas used in the percutaneous placements of VVMS were smaller than recommended in the 2022 ELSO Handbook (6), but oxygen saturation at 24 hours ECMO flows were effective. A larger study would be required to further explore the above findings.

## Supplementary Material


