# Supplementary material for: Multisite Venovenous Extracorporeal Membrane Oxygenation in Pediatric Patients Under 15 kg: Three-Center, Analysis of Surgical Versus Percutaneous Approach and Thrombosis Risk, 2017–2024
Source: Pediatr Crit Care Med. 2025 Nov 7;27(1):25–32. doi: 10.1097/PCC.0000000000003858 (PMC12771976; doi:10.1097/PCC.0000000000003858)
Supplement: Supplementary file 1 [file pcc-27-25-s001.pdf]

Clinical Guidance

Paediatric Critical Care:
ECMO Unfractionated Heparin Anticoagulation

Summary
Clinical guideline for the management of routine anticoagulation using unfractionated heparin for patients on ECMO on the Evelina site. This guideline is not be used to manage patients who are receiving anticoagulation for other purposes.

Document Detail	
Document type	Clinical Guideline
Document name	Paediatric Critical Care: ECMO Unfractionated Heparin Anticoagulation
Document location	GTi Clinical Guidance Database
Version	V2.0
Effective from	*June 2024*
Review date	*June 2027*
Owner	Head of Service, PICU
Author(s)	Andrew Nyman, Jon Lillie (PICU Consultant)
Approved by, date	Thrombosis & Thromboprophylaxis Committee Dec 19th 2018<br>Evelina London Guideline Committee, Jan 2019
Superseded documents	
Related documents	Paediatric Critical Care: Extracorporeal Membrane Oxygenation(ECMO): Bleeding and Blood Product Use
Keywords	ECMO, extracorporeal membrane oxygenation, VA, VV, unfractionated, heparin, anticoagulation, bleeding, FFP, thrombin, anti, Xa, child, Evelina
Relevant external law, regulation, standards	

Change History		
Date	Change details, since approval	Approved by
06/24	Anti Xa sampling changed to reflect now routine trust test: check 4 h after changes and no need to send on ice or call lab. Checking ACT prior to VA ECMO added at surgical request	

Glossary: ACT: activated clotting time, CVVH: continuous veno-venous hemofiltration, ECMO: extracorporeal membrane oxygenation.

Paediatric Critical Care
ECMO Unfractionated Heparin Anticoagulation

For routine anticoagulation for children on ECMO. If bleeding see bleeding guidance.

Initiation of unfractionated (UF) heparin anticoagulation

Acute:

- UF Heparin at cannulation = 75units/kg bolus (surgical instruction), may repeat if >30 min elapses to cannulation. If VA ECMO where stroke risk is higher- ideally confirm heparin effect ACT>200 prior to cannulation of artery.
- Standard UF heparin concentration **1mL/h = 25 units/kg/h** (1250units UF heparin x wt (kg) in 50mL 0.9% sodium chloride)
- Start infusion at 25 units/kg/h after cannulation (without ACT/coagulation tests) unless post bypass (see below) or bleeding is significant.

Post cardiosurgical bypass:

- Start heparin infusion at 25 units/kg/h when ACT < 300 seconds. Rarely require bolus dose.

Key points:

- Adjust UF heparin infusion in increments of **5units/kg/h** if change in anti-coagulation needed – Do not routinely bolus. In some patients adjustments of less than 5units/kg/h are required to achieve target anti-Xa levels.
- In certain settings e.g. severe pulmonary haemorrhage the UF heparin dosage/ infusion may be held or altered
- If patient on ECMO requires CVVH then no extra UF heparin is required for the CVVH circuit including prime
- Anti-Xa levels should be **done after 4 hours on ECMO** and usually **once per shift** or more frequently following any significant changes in patient / circuit bleeding or thrombosis. They should be paired with coag tests (INR, APTT, Fib).

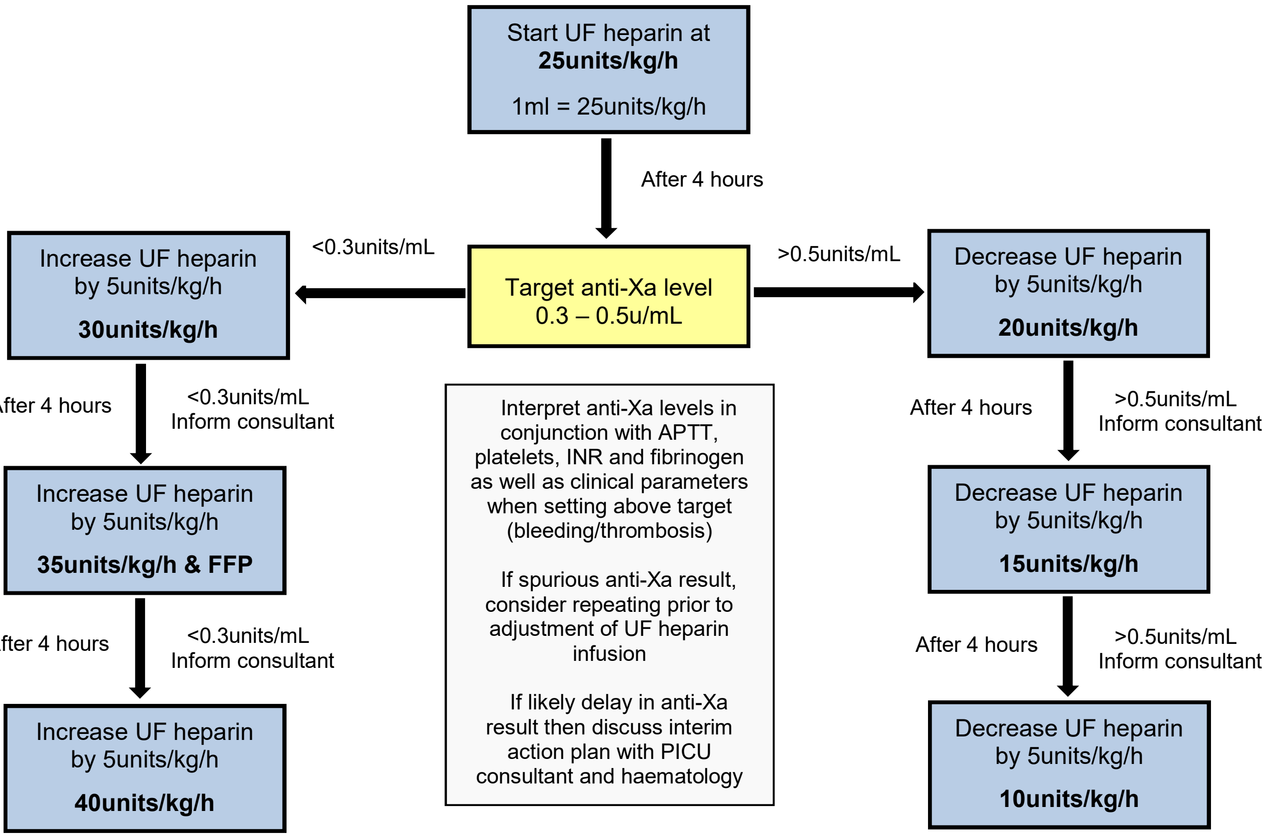

- Ensure antithrombin levels >60%, if less <60% give antithrombin concentrate
- Call paediatric haematology SpR who will supply and advise on dose
- FFP can be used but effects are variable
- Increase UF heparin by 5units/kg/h every 12hours to max 50units/kg/h – discuss with ECMO team and haematology

- Repeat anti-Xa level after 4 hours
- Decision to reduce UF heparin to 10units/kg/h or stopping UF heparin is the decision of the ECMO team

Semmelweis Anticoagulation - ECMO
Version 1.2
2023.10.06 by Lodi Csaba, Szasz Barbara

Prime: 500 U heparin/1000 ml Saline

Bolus heparin 75 U/kg when cannulated

After initiation of ECMO:
- ACT every 60 minutes
- when ACT ‹ 200 Heparin is started at a dose of 25 U/kg/h without a bolus

4 hours after the start of treatment:
- full blood count
- basic clotting (incl. fibrinogen)
- anti-Xa
- antithrombin
- Clot-pro

<u>**Anti-Xa** target range:</u>
No clots: 0.3-0.5
Clot in system: 0,5-0,7 or higher

No clots:
Heparin modification based on anti-Xa:
‹ 0,1 increase by 10E/kg/hr
0,1-0,3 increase by 5E/kg/hr
0,3-0,5 No changes
0,5-0,7 reduce by 5E/kg/hr
›0,7reduce by 10E/kg/hr

Clot in the system:
Heparin modification based on anti-Xa:
‹0,1increase by 10E/kg/hr
0,1-0,3 increase by 10E/kg/hr
0,3-0,5 increase by 10E/kg/hr
0,5-0,7 No changes
0,7-0,9 reduce by 5E/kg/hr
›0,9reduce by 10E/kg/hr

Laboratory control is required after 6 hours of dose modification.

<u>**Antithrombin** and anti-Xa</u>
Antithrombin target range ›50

Antithrombin correction is not performed until the dose of heparin reaches 40 U/kg/h.

Clot in the system	*anti-Xa level*	*AT*	*change*
yes	0,5-0,7	‹50	anti-Xa after 4 hours
yes	‹0,5	‹40	Administration of 100-150E/kg AT
yes	‹0,5	40-50	20ml/kg FFP
no	0,3-0,5	‹50	anti-Xa after 4 hours
no	‹0,3	‹50	Heparin modification see above

Timing of tests during the first 24 hours:

- ACT per hour
- blood count, INR, PTT, fibrinogen, AT, anti-Xa every 4 hours

Timing of tests after 24 hours:

- ACT every 4 hours
- INR, PTT, fibrinogen, anti-Xa every 8 hours
- AT, blood count every 12-24 hours

If the patient remains stable, there are no haemorrhagic or thrombotic complications:

- ACT, INR, APTI, fibrinogen, anti-Xa every 12 hours
- AT, blood count every 24 hours

Note: 4 hours after FFP and AT administration there should be anti-Xa control
After a haemorrhagic or thrombotic event, emergency tests are necessary!

Platelet		> 100,000	2-4 E Low Volume Thr
Hematocrit		> 35%	15 ml/kg filtered, cross matched RC
Fibrinogen	no bleeding	> 1 g/dl	
	haemorrhage	> 1,5 g/dl	
INR	no bleeding	< 3	FFP 15 ml/kg
	haemorrhage	< 1.5	FFP 15 ml/kg
Antithrombin		>50%	

Algorithm for discrepant lab values

a-Xa / aPTT	<60 sec	60-90 sec	>90 sec
<0.3	heparin↑	heparin↑	?factor deficiency – ? FFP
0.3-0.5	?thrombophilia	no change	repeat lab - ? FFP
>0.5	Unlikely, repeat lab	?thrombophilia, repeat lab	heparin↓

During ECMO treatment, it is mandatory to be available in the department:

- crossmatched blood: 1 U and 2x1/4 U RC
- topical hemostatic: Spongostan, Surgicell

Available in the hospital:
- compatible FFP: 4x1/4 U
- Fibrinogen – Haemocomplettan
- Factor preparation – Prothromplex Total
- Exacyl

Heim Pál Anticoagulation Protocol (2020)

- Pre cannulation tests: INR, fibrinogen, aPTT, AT-III, D-dimer
  Correction of preexisting coagulopathies
- 2 IU/ml Na-heparin in priming solution
- Before cannulation: 50-100 IU/kg Na-heparin bolus
  - Target ACT>200 s
- Start of heparin pump 2 hours after cannulation: 10-20 IU/kg/hour
- Monitoring:
  - aPTT
    - 2 hours after adjusting heparin
    - every 6 hours in steady state
  - Daily (or if needed): INR, fibrinogen, D-dimer, antithrombin-III

APTT guided heparin dosage

	Standard risk			High thrombotic risk			High bleeding risk		
Target APTT	60-85 s			85-120 s			40-60 s		
	APTT (s)	bolus (IU)	adjustment (IU/h)	APTT (s)	bolus (IU)	adjustment (IU/h)	APTT (s)	bolus (IU)	adjustment (IU/h)
	<50	0	+20%	<60	0	+20%	<40	0	+10%
	50-59	0	+10%	60-85	0	+10%	40-60	0	0
	60-85	0	0	86-120	0	0	60-90	0	-10%
	86-120	0	-10 %	120-150	0	-10%	>90%	0	+20%
	>120	0	-20 %	>150	0	-20%			

Target hgb: 100g/l
Target platelet: 100 (lower in haematologic patients)
Target fibrinogen: >1 g/l
Target antithrombin-III: >35%

Stopping heparin at least 2 hours before decannulation
Prophylactic enoxaparine till central line in situ
